# Migration of a double j stent presenting as a painless total urinary incontinence

**DOI:** 10.11604/pamj.2019.33.270.15809

**Published:** 2019-07-30

**Authors:** Youness Jabbour, Youness Boukhlifi

**Affiliations:** 1 Urology B Department, Ibn Sina Teaching Hospital, Rabat, Morocco; 2 Faculty of Medicine and Pharmacy, Mohammed V University, Rabat, Morocco; 3 Urology Department, Military Hospital of Instruction Mohammed V, Rabat, Morocco

**Keywords:** Urinary incontinence, double j stent, ureteric stent migration

## Image in medicine

Double j (DJ) stent is a thin hollow tube placed inside the ureter to ensure drainage of urine from the kidney into the bladder. It holds its denomination of the presence of J shaped curls at both ends to keep the tube in place and prevent migration. Urinary Incontinence is a frequent complaint of patients holding DJ stents mostly presenting as urge or stress incontinence. Stent-related total incontinence is the result of stent migration across the urethral sphincter making it ineffectif. It's about a 35-year-old man who undergone a flexible laser ureteroscopy for management of an obstructive calculi of the lumbar ureter with DJ stent placement. It was planned to remove the DJ stent after one month, but it appears that the patient tolerated his DJ stent so well that he didn't show up until three months later with total urinary incontinence. He reported no incidents after stent placement but an onset of a sudden total and painful urinary incontinence without accompanying signs evolving for two days. Since the patient was known to be a DJ stent holder and given the sudden onset of the urinary incontinence, we thought about the migration of the DJ stent through the uretral sphincter. Urethro-cystoscopy showed the presence of the lower loop of the double j stent at the anterior ureter and allowed the extraction of the double j stent. The patient regained his continence immediately after removal of the double-j stent and left the hospital the same day.

**Figure 1:**
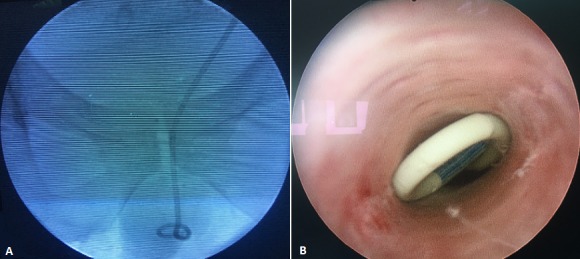
A) X-ray showing the lower loop of the double j stent out of the bladder; B) uretroscopy showing the lower loop of the double j stent at the anterior ureter

